# Contemporary Evaluation of Tebipenem *In Vitro* Activity against Enterobacterales Clinical Isolates Causing Urinary Tract Infections in US Medical Centers (2019–2020)

**DOI:** 10.1128/spectrum.02057-22

**Published:** 2023-01-10

**Authors:** Rodrigo E. Mendes, S. J. Ryan Arends, Jennifer M. Streit, Ian Critchley, Nicole Cotroneo, Mariana Castanheira

**Affiliations:** a JMI Laboratories, North Liberty, Iowa, USA; b Spero Therapeutics, Cambridge, Massachusetts, USA; Univ of Texas Southwestern Medical Center

**Keywords:** resistance, surveillance, CTX-M, Gram-negative bacteria

## Abstract

Tebipenem pivoxil is an oral broad-spectrum carbapenem. This study evaluated the activity of tebipenem and comparators against UTI Enterobacterales from US hospitals (2019–2020). 3,576 Enterobacterales causing UTI in 52 centers in 9 US Census Divisions were included. Susceptibility testing followed the CLSI broth microdilution method. Escherichia coli, Klebsiella pneumoniae, and Proteus mirabilis with an MIC of ≥2 μg/mL for ceftazidime, ceftriaxone, and/or aztreonam were designated ESBL. Isolates were also grouped based on MDR phenotype. Tebipenem, meropenem, and ertapenem had MIC_90_ against Enterobacterales of 0.06 μg/mL, 0.06 μg/mL and 0.03 μg/mL, respectively. Low susceptibility results for aztreonam (87.1% susceptible), cefazidime (88.1%), ceftriaxone (84.8%), and other agents were observed. Tebipenem and ertapenem were equally potent (MIC_90_, 0.015 to 0.03 μg/mL) against E. coli and K. pneumoniae, whereas ertapenem showed an MIC 8-fold lower than tebipenem against P. mirabilis. Oral agents, such as amoxicillin-clavulanate, levofloxacin, and trimethoprim-sulfamethoxazole, showed elevated nonsusceptibility rates in the Middle Atlantic region (26, 45, 47, and 41%, respectively). ESBL prevalence varied from 7% to 16%, except in the Middle Atlantic region (42%). The carbapenems were active against ESBL and MDR isolates (93.7 to 96.8% susceptible). Elevated rates of ESBL in UTI pathogens in US hospitals were noted as well as a uniform in vitro potency (MIC_90_) of tebipenem and the intravenous carbapenems, regardless of phenotype.

**IMPORTANCE** The occurrence of urinary-tract Enterobacterales pathogens producing ESBL enzymes in community and nosocomial settings continues to increase, as does the coresistance to fluoroquinolones, trimethoprim-sulfamethoxazole and nitrofurantoin often exhibited by these pathogens. This scenario complicates the clinical empirical and guided management of UTI by precluding the use of oral and many intravenous options. Oral options appear compromised even among some ESBL-negative isolates, against which the use of parenteral agents may be required. In addition, the interregional variability of susceptibility results of US UTI pathogens provides a less predictable susceptibility pattern to inform empirical treatment decisions. This study evaluated the *in vitro* activity of tebipenem against contemporary uropathogens, including those resistant to currently available oral options.

## INTRODUCTION

Urinary tract infections (UTIs) remain a public health problem in both community and hospital settings. UTIs represent the fifth most common type of health care-associated infection, with an estimated 62,700 infections requiring treatment in acute care hospitals in the United States (1). Escherichia coli, Klebsiella pneumoniae, and Proteus mirabilis are the most frequent Gram-negative bacteria implicated in UTIs. Moreover, according to the Centers for Disease Control and Prevention report on antimicrobial resistance in 2019, approximately 197,400 Enterobacterales with an ESBL phenotype are detected each year in the United States (2, 3). This report also showed a consistent increase in the ESBL phenotype among Enterobacterales between 2012 and 2017. Other studies also reported increasing rates of ESBL pathogens causing UTI in hospitals and community settings (4–6).

In general, rates of resistance to first-line oral options clinically available for the treatment of complicated UTI (cUTI) and pyelonephritis, such as the fluoroquinolones and trimethoprim-sulfamethoxazole, exceeds 20% in the USA. These rates increase to ≥60% against ESBL isolates (7). This scenario has resulted in dramatic increases in emergency department visits and hospital admissions for UTIs, especially due to the need for intravenous agents given the lack of effective oral options (8, 9). Also, the increase of resistance phenotypes has led to delays of effective therapy, longer hospital stays, and higher hospitalization costs (10–12).

Tebipenem is a broad-spectrum agent with *in vitro* and *in vivo* activity against a variety of clinically important Gram-negative and Gram-positive pathogens, including ESBL-producing Enterobacterales (13). This orally bioavailable carbapenem is currently under clinical development for cUTI, including pyelonephritis. In a large phase 3 clinical trial (ADAPT-PO), oral tebipenem (600 mg every 8 h) was shown to be noninferior to intravenous ertapenem (1 g every 24 h) (12). This study evaluated the *in vitro* activity of tebipenem and the comparator agents that are commonly used for the treatment of UTI against Enterobacterales collected from US patients in 2019 to 2020 as part of the STEWARD Surveillance Program.

## RESULTS

E. coli (*n *=* *2,339; 65.4%) represented the majority of Enterobacterales associated with UTI in this study, followed by K. pneumoniae (*n *=* *511; 14.3%) and P. mirabilis (*n *=* *235; 6.6%). Other pathogens comprised 24 other species or species groups (*n *=* *491; 13.7%) (Table 1).

**TABLE 1:** *In vitro* activity of tebipenem against Enterobacterales clinical isolates causing UTI in US hospitals

Organism/group (no. of isolates)	No. and cumulative % of isolates inhibited at an MIC (μg/mL)													MIC_50_	MIC_90_
	≤0.004	0.008	0.015	0.03	0.06	0.12	0.25	0.5	1	2	4	8	>8		
All^*a*^ (3,576)	4 (0.1)	606 (17.1)	2,060 (74.7)	429 (86.7)	207 (92.4)	193 (97.8)	50 (99.2)	9 (99.5)	0 (99.5)	3 (99.6)	2 (99.6)	5 (99.8)	8 (100)	0.015	0.06
E. coli (2,339)	4 (0.2)	578 (24.9)	1,578 (92.3)	134 (98.1)	29 (99.3)	10 (99.7)	4 (99.9)	1 (>99.9)	0 (>99.9)	0 (>99.9)	1 (100.0)			0.015	0.015
K. pneumoniae (511)		3 (0.6)	296 (58.5)	176 (93.0)	18 (96.5)	3 (97.1)	2 (97.5)	1 (97.7)	0 (97.7)	1 (97.8)	1 (98.0)	4 (98.8)	6 (100)	0.015	0.03
P. mirabilis (235)		1 (0.4)	6 (3.0)	23 (12.8)	63 (39.6)	120 (90.6)	22 (100.0)							0.12	0.12
Non-ESBL (2,643)^*b*^	4 (0.2)	560 (21.3)	1,611 (82.3)	238 (91.3)	88 (94.6)	120 (99.2)	22 (100)							0.015	0.03
ESBL (442)^*c*^		22 (5.0)	269 (65.8)	95 (87.3)	22 (92.3)	13 (95.2)	6 (96.6)	2 (97.1)	0 (97.1)	1 (97.3)	2 (97.7)	4 (98.6)	6 (100)	0.015	0.06
MDR (222)^*d*^		14 (6.3)	134 (66.7)	45 (86.9)	8 (90.5)	10 (95.0)	4 (96.8)	0 (96.8)	0 (96.8)	1 (97.3)	0 (97.3)	1 (97.7)	5 (100)	0.015	0.06

a Includes Citrobacter amalonaticus*/farmeri* (4), C. freundii species complex (57), *C. koseri* (40), Enterobacter asburiae (1), E. cloacae species complex (143), *E. hormaechei* (1), Escherichia coli (2,339), *E. marmotae* (1), Hafnia alvei (2), Klebsiella aerogenes (58), *K. ornithinolytica* (1), K. oxytoca (87), K. pneumoniae (511), *K. variicola* (5), Morganella morganii (21), Proteus hauseri (1), P. mirabilis (235), *P. penneri* (2), P. vulgaris group (13), Providencia rettgeri (6), P. stuartii (4), Raoultella ornithinolytica (1), *R. planticola* (2), Serratia fonticola (1), S. liquefaciens (1), S. marcescens (36), *Citrobacter* spp. (1), and *Raoultella* spp. (2).

b Includes 1,988 E. coli, 430 K. pneumoniae, and 225 P. mirabilis displaying MIC results of <2 μg/mL for ceftazidime, aztreonam, and ceftriaxone, which were presumptively characterized here as isolates absent of ESBL enzymes.

c Includes 351 E. coli, 81 K. pneumoniae, and 10 P. mirabilis displaying MIC results of ≥2 μg/mL for ceftazidime, aztreonam and/or ceftriaxone, which were presumptively characterized here as ESBL producers.

d Includes 175 E. coli, 40 K. pneumoniae, and 7 P. mirabilis displaying MIC results of ≥2 μg/mL for ceftazidime, aztreonam and/or ceftriaxone, which were presumptively characterized here as ESBL producers. These isolates also were not susceptible to levofloxacin (MIC, ≥1 μg/mL) and trimethoprim-sulfamethoxazole (≥4/76 μg/mL).

Overall, the national rate of E. coli, K. pneumoniae, and P. mirabilis showing an ESBL phenotype was 14.3%. Rates between 7% and 11% were detected in East North Central, West North Central, West South Central, and South Atlantic regions. Higher rates were noted in the Pacific (14.5%), Mountain (15.4%), East South Central (16.3%), and Middle Atlantic (42%) regions (Table 2). Similar proportions of E. coli (15.0%) and K. pneumoniae (15.9%) met the MIC criteria for an ESBL phenotype, and a smaller prevalence was observed in P. mirabilis (4.3%) (Table 1). Tebipenem, ertapenem, and meropenem showed similar activity against all Enterobacterales, with MIC_90_ values of 0.06 μg/mL, 0.03 μg/mL, and 0.06 μg/mL, respectively, and high overall rates of susceptibility to the comparator carbapenems ertapenem (98.5%) and meropenem (99.6–99.7%) (Table 3). Piperacillin-tazobactam (MIC_90_, 8 μg/mL) showed susceptibility results of 92.9% against all isolates (Table 3).

**TABLE 2:** Nonsusceptibility rates for amoxicillin-clavulanate, levofloxacin, and trimethoprim-sulfamethoxazole against most common isolates (E. coli, K. pneumoniae, and P. mirabilis) causing UTI by Census Divisions

Region	% not susceptible^*a*^					ESBL^*b*^
	A-C		Levofloxacin		TMP-SMX	
All	17.0		22.7		28.5	14.3
New England	13.7		15.8		23.2	9.9
Middle Atlantic	26.4		46.7		41.4	41.9
East North Central	15.3		20.8		23.7	10.5
West North Central	14.2		14.4		24.4	6.9
South Atlantic	15.0		20.5		29.5	10.8
East South Central	19.9		23.3		22.4	16.3
West South Central	17.2		25.3		34.5	10.7
Mountain	20.5		22.8		34.9	15.4
Pacific	14.9		17.9		25.8	14.5

a Includes 2,339 E. coli, 511 K. pneumoniae, and 235 P. mirabilis; % of isolates not susceptible according to CLSI 2021; A-C, amoxicillin-clavulanate; TMP-SMX, trimethoprim-sulfamethoxazole.

b Includes 351 E. coli, 81 K. pneumoniae, and 10 P. mirabilis distributed across 9 US Census Divisions displaying MIC results of ≥2 μg/mL for ceftazidime, aztreonam and/or ceftriaxone, and presumptively characterized here as ESBL producers.

**TABLE 3:** Antimicrobial activity of tebipenem and comparator agents against Enterobacterales and phenotypic subsets^*a*^

Antimicrobial agent	MIC (μg/mL)			CLSI^*b*^		
	50%	90%	Range	%S	%I	%R
All (3,576)						
Tebipenem	0.015	0.06	≤0.004 to >8	-	-	-
Amoxicillin-clavulanic acid	4	32	≤0.25 to >32	76.1	10.9	13.1
Aztreonam	0.12	16	≤0.03 to >16	87.1	2.0	10.9
Cefazolin	2	>32	≤0.5 to >32	-	-	-
Ceftazidime	0.25	8	≤0.015 to >32	88.1	2.1	9.8
Ceftriaxone	≤0.06	>8	≤0.06 to >8	84.8	0.3	14.9
Cefuroxime	4	>64	≤0.5 to >64	-	-	-
Ertapenem	≤0.008	0.03	≤0.008 to >2	98.5	0.8	0.7
Levofloxacin	0.06	16	≤0.015 to >32	79.4	2.2	18.4
Meropenem	≤0.015	0.06	≤0.015 to >32	99.6	0.1	0.3
Piperacillin-tazobactam	2	8	≤0.06 to >128	92.9	2.7	4.4
Trimethoprim-sulfamethoxazole	≤0.12	>4	≤0.12 to >4	73.9		26.1
Non-ESBL (2,643)						
Tebipenem	0.015	0.03	≤0.004 to 0.25	-	-	-
Amoxicillin-clavulanic acid	4	16	≤0.25 to >32	89.4	8.5	2.1
Aztreonam	0.06	0.12	≤0.03 to 1	100.0	0.0	0.0
Cefazolin	2	8	≤0.5 to >32	-	-	-
Ceftazidime	0.12	0.25	0.03 to 1	100.0	0.0	0.0
Ceftriaxone	≤0.06	0.12	≤0.06 to 1	100.0	0.0	0.0
Cefuroxime	4	8	≤0.5 to 32	95.9^*c*^	3.6	0.5
Ertapenem	≤0.008	0.015	≤0.008 to 0.25	100.0	0.0	0.0
Levofloxacin	0.03	8	≤0.015 to >32	85.4	1.6	13.0
Meropenem	≤0.015	0.03	≤0.015 to 0.25	100.0	0.0	0.0
Piperacillin-tazobactam	2	4	≤0.06 to >128	98.4	0.6	1.0
Trimethoprim-sulfamethoxazole	≤0.12	>4	≤0.12 to >4	77.9		22.1
ESBL (442)						
Tebipenem	0.015	0.06	0.008 to >8	-	-	-
Amoxicillin-clavulanic acid	16	>32	2 to >32	44.6	32.4	23.1
Aztreonam	>16	>16	0.12 to >16	18.8	14.7	66.5
Cefazolin	>32	>32	8 to >32	-	-	-
Ceftazidime	16	>32	0.12 to >32	26.7	15.6	57.7
Ceftriaxone	>8	>8	0.12 to >8	6.6	1.1	92.3
Cefuroxime	>64	>64	4 to >64	2.5^*c*^	2.5	95.0
Ertapenem	0.03	0.25	≤0.008 to >2	93.8	2.8	3.5
Levofloxacin	8	32	≤0.015 to >32	28.4	5.2	66.4
Meropenem	0.03	0.06	≤0.015 to >32	97.3	1.1	1.6
Piperacillin-tazobactam	4	32	≤0.06 to >128	74.9	12.7	12.4
Trimethoprim-sulfamethoxazole	>4	>4	≤0.12 to >4	33.5		66.5
MDR (222)						
Tebipenem	0.015	0.06	0.008 to >8	-	-	-
Amoxicillin-clavulanic acid	16	32	2 to >32	41.4	39.2	19.4
Aztreonam	>16	>16	0.12 to >16	15.8	15.8	68.5
Cefazolin	>32	>32	32 to >32	-	-	-
Ceftazidime	16	>32	0.12 to >32	24.8	17.6	57.7
Ceftriaxone	>8	>8	0.25 to >8	1.8	1.4	96.8
Cefuroxime	>64	>64	4 to >64	1.4^*c*^	0.5	98.2
Ertapenem	0.03	0.25	≤0.008 to >2	94.0	2.8	3.2
Levofloxacin	16	32	1 to >32	0.0	8.6	91.4
Meropenem	0.03	0.06	≤0.015 to >32	96.8	0.9	2.3
Piperacillin-tazobactam	4	32	≤0.06 to >128	68.9	16.7	14.4
Trimethoprim-sulfamethoxazole	>4	>4	4 to >4	0.0		100.0

a See the footnote on Table 1 for a description of all 3,576 species of Enterobacterales included in this study. The non-ESBL category includes 1,988 E. coli, 430 K. pneumoniae, and 225 P. mirabilis displaying MIC results of <2 μg/mL for ceftazidime, aztreonam, and ceftriaxone, and these isolates are presumptively characterized here as absent of ESBL enzymes. The ESBL phenotype were those E. coli (351), K. pneumoniae (81), and P. mirabilis (10) displaying MIC results of ≥2 μg/mL for ceftazidime, aztreonam, and/or ceftriaxone. The MDR phenotype were those isolates presumptively characterized as ESBL producers that also were not susceptible to levofloxacin (MIC, ≥1 μg/mL) and trimethoprim-sulfamethoxazole (≥4/76 μg/mL).

b Criteria as published by CLSI (2022); “-“, breakpoint not available or not available for any given species included within a group (i.e., cefazolin).

c Using parenteral breakpoints.

Tebipenem and ertapenem were equally potent (MIC_90_, 0.015 to 0.03 μg/mL) against E. coli and K. pneumoniae, whereas ertapenem (MIC_50/90_, 0.015/0.015 μg/mL) showed MIC results 8-fold lower than tebipenem (MIC_50/90_, 0.12/0.12 μg/mL) against P. mirabilis (Table 3).

When evaluating the activity of oral agents against the most common species of E. coli, K. pneumoniae, and P. mirabilis, 17% to 29% of these isolates were not susceptible to amoxicillin-clavulanate (17.0%), levofloxacin (22.7%), and trimethoprim-sulfamethoxazole (28.5%), according to CLSI breakpoints (Table 2). However, a great percentage of isolates (26 to 47%) that were not susceptible to these oral options tended to originate from centers located in the Middle Atlantic (Table 2). Against non-ESBL E. coli, K. pneumoniae, and P. mirabilis isolates, amoxicillin-clavulanate, levofloxacin, and trimethoprim-sulfamethoxazole had susceptibility results between 77.9% and 89.4% (Table 3).

When tested against the isolates that met the MIC criteria for ESBL or MDR phenotypes, tebipenem demonstrated MIC_50_ results of 0.015 μg/mL, which were equivalent to those obtained against the non-ESBL group (Table 1). In contrast, the ertapenem MIC results obtained against the ESBL (MIC_50/90_, 0.03/0.25 μg/mL) and MDR (MIC_50/90_, 0.03/0.25 μg/mL) groups of isolates were higher than those obtained against their non-ESBL counterparts (MIC_50/90_, ≤0.008/0.015 μg/mL). In general, the carbapenems (93.8 to 97.3% susceptible) had elevated susceptibility results against isolates exhibiting ESBL and/or MDR phenotypes, while piperacillin-tazobactam had the highest results (68.9 to 74.9% susceptible) among noncarbapenem agents (Table 3).

## DISCUSSION

This study described the activity of tebipenem and other comparator agents tested against Enterobacterales causing UTI in US hospitals. Against all Enterobacterales, carbapenems demonstrated the best activity, with resistance rates of 0.3% to 0.7%, whereas resistance to oral agents and cephalosporins was observed more frequently at rates of 9.8% to 26.1%. These results reflect the continuous dissemination of the ESBL phenotype in E. coli, K. pneumoniae, and P. mirabilis isolates in the US during the last 2 decades (14). Rates of ESBL production among E. coli, K. pneumoniae, and P. mirabilis causing various infections in US hospitals were reported to be between 12% and 16% during 2013 to 2016 (11, 14, 15). A similar collection of E. coli and K. pneumoniae that caused UTI from the 2016 SENTRY program for the US revealed ESBL rates of 12% and 11%, respectively (15). The results presented here showed presumptive ESBL rates of approximately 15% in E. coli and 16% in K. pneumoniae. These latest conclusions indicate that the dissemination of ESBL isolates causing UTI in US hospitals continues, as does the coresistance often exhibited by these pathogens (Table 3).

UTI caused by ESBL pathogens often precludes the use of oral and many intravenous options for empirical or guided treatment. Additionally, 11% to 22% of non-ESBL isolates were also not susceptible to oral options, such as amoxicillin-clavulanate, levofloxacin, and trimethoprim-sulfamethoxazole. Therefore, these results show the need for an orally bioavailable agent that could provide broad spectrum coverage for the empirical and guided management of UTI in both inpatient and ambulatory care settings, including infections caused by ESBL and/or MDR isolates.

It is important to emphasize that, upon approval, the clinical use of tebipenem should be exercised judiciously. Tebipenem has an intrinsic broad spectrum of activity, which resembles the activity of intravenous carbapenems (13). The convenience provided by such an oral agent could potentially exacerbate its clinical utilization, which could encourage the emergence and dissemination of isolates carrying carbapenemase genes. Stewardship programs are critical to minimize indiscriminate use of antimicrobial agents.

This study evaluated the *in vitro* activity of tebipenem and comparator agents against UTI pathogens of various phenotypes. However, due to a lack of further patient information, a distinction between isolates causing complicated or uncomplicated UTI could not be made. In addition, a specific analysis of tebipenem activity based on molecular data were not performed here, but one has been published elsewhere against subsets of E. coli (16). This study reported on the high rates of ESBL and MDR rates in UTI pathogens in US hospitals and the uniform *in vitro* potency (MIC_90_) of the oral tebipenem and intravenous carbapenems, where other oral treatment options did not seem to provide acceptable (i.e., >90% susceptible) *in vitro* susceptibility rates. These data benchmark the *in vitro* activity of tebipenem tested against Enterobacterales. Since tebipenem is being developed as an oral prodrug, it could offer an additional and convenient tool for managing UTI caused by Enterobacterales when approved for clinical use.

## MATERIALS AND METHODS

### Clinical isolates.

A total of 3,576 Enterobacterales isolates were recovered from patients with UTI in 52 medical centers located in 9 US Census Divisions during 2019 and 2020. These isolates were included in the STEWARD Surveillance Program. STEWARD mirrored the design of the SENTRY Antimicrobial Surveillance Program, which is a cross-sectional, laboratory-based program that surveys the frequency of organisms causing various community- and hospital-associated infections, observes antimicrobial susceptibility patterns, and monitors for the emergence of resistance (17). Participating laboratories followed specific instructions for selecting consecutive and unique isolates (1 isolate per patient per infection episode). Only isolates deemed to be clinically relevant and responsible for UTI based on local criteria (e.g., ≥100,000 CFU/mL) were selected until a target number was reached. The sites initially performed the bacterial identifications, which were later confirmed by JMI Laboratories using matrix-assisted laser desorption ionization-time of flight mass spectrometry (Bruker Daltonics, Bremen, Germany).

### Antimicrobial susceptibility testing.

Susceptibility testing was performed using the Clinical and Laboratory Standards Institute (CLSI) broth microdilution method (18). Frozen-form broth microdilution panels were manufactured by JMI Laboratories (North Liberty, IA, USA) and contained cation-adjusted Mueller-Hinton broth. Panels were quality assured both before and during use according to CLSI guidelines (18, 19). Agents presented here were those included in the surveillance program for tebipenem. These agents were not chosen based on indication (i.e., cUTI), but rather to characterize the susceptibility profiles of included species and group of species (e.g., ESBL or carbapenemases). MIC interpretations for comparator agents followed CLSI breakpoint criteria, as available (19). E. coli, K. pneumoniae, and P. mirabilis with MIC values of ≥2 μg/mL for ceftazidime, ceftriaxone, and/or aztreonam were phenotypically characterized as ESBL. Isolates were also grouped as an MDR phenotype if an ESBL phenotype was present and the isolate was nonsusceptible to fluoroquinolone (levofloxacin) and trimethoprim-sulfamethoxazole using CLSI breakpoints (19).
